# Green Synthesis and Comparative Analysis of Silver, Copper Oxide, and Bimetallic Ag/CuO Nanoparticles Using *Cistus creticus* L. Extract: Physicochemical Properties, Stability, and Antioxidant Potential

**DOI:** 10.3390/ijms26062518

**Published:** 2025-03-11

**Authors:** Chrysi Chaikali, Nicole Dora Stola, Paraskevi Lampropoulou, Dimitrios Papoulis, Fotini N. Lamari, Malvina Orkoula, Michail Lykouras, Konstantinos Avgoustakis, Sophia Hatziantoniou

**Affiliations:** 1 Laboratory of Pharmaceutical Technology, Department of Pharmacy, School of Health Sciences, University of Patras, 26504 Patras, Greece; chem3813@ac.upatras.gr (C.C.); up1022689@upnet.gr (N.D.S.); avgoust@upatras.gr (K.A.); 2 Department of Geology, University of Patras, 26504 Patras, Greece; p.lampropoulou@upatras.gr (P.L.); papoulis@upatras.gr (D.P.); 3 Laboratory Pharmacognosy & Chemistry of Natural Products, Department of Pharmacy, School of Health Sciences, University of Patras, 26504 Patras, Greece; flam@upatras.gr; 4 Laboratory of Instrumental Pharmaceutical Analysis, Department of Pharmacy, School of Health Sciences, University of Patras, 26504 Patras, Greece; malbie@upatras.gr; 5 Institute of Chemical Engineering Sciences, Foundation of Research and Technology-Hellas (ICE-HT/FORTH), 26504 Patras, Greece; lykouras@iceht.forth.gr

**Keywords:** green synthesis, *Cistus creticus* L. extract, silver nanoparticles, copper oxide nanoparticles, bimetallic nanoparticles, antioxidant

## Abstract

This study investigates silver (Ag), copper oxide (CuO), and bimetallic Ag/CuO nanoparticles (NPs) synthesized using *Cistus creticus* L. extract, focusing on their synthesis, physicochemical characteristics, and antioxidant activity. Green synthesis methods utilizing plant extracts offer environmentally benign routes for nanoparticle fabrication, attracting significant interest across multiple fields. NP formation was confirmed by UV/Vis and total X-ray fluorescence (TXRF) spectroscopy, while dynamic and electrophoretic light scattering (DLS, ELS) characterized particle size and ζ-potential, respectively. AgNPs exhibited the smallest particle size (30.8 ± 8.81 nm), while CuONPs had the largest (44.07 ± 19.19 nm). For Ag/CuONPs, the ζ-potential value was −77.9 ± 2.99 mV. Morphological and structural analyses performed using transmission electron microscopy (TEM), selected area electron diffraction (SAED), and X-ray diffraction (XRD) revealed that AgNPs were spherical, while CuONPs and Ag/CuONPs exhibited spherical and polymorphic structures. Colloidal stability studies over 60 days demonstrated that the NPs were highly stable, indicating their suitability for pharmaceutical and cosmetic applications. Antioxidant activity, assessed via the DPPH assay, demonstrated that CuONPs had the highest free radical scavenging activity. By systemically comparing Ag, CuO, and bimetallic Ag/CuONPs synthesized from *Cistus creticus* L. extract, this study provides valuable insights for the development of tailored nanomaterials with diverse applications in pharmaceutics and cosmetics.

## 1. Introduction

Metallic nanoparticles (NPs) have gained considerable attention due to their unique physicochemical properties, which differ significantly from their bulk counterparts. These properties arise from their high surface-area-to-volume ratio and are strongly influenced by their size and shape [1].

Among metallic NPs, silver (Ag) and copper (Cu) nanoparticles are particularly notable for their antimicrobial, anticancer, and antioxidant activities. Due to these characteristics, particularly their antimicrobial activity, AgNPs, CuNPs, and copper oxide NPs (CuONPs) have been widely used in medical applications such as dental materials, wound dressing bond implants, and antibiofilm treatments [2,3,4].

The combination of Ag and Cu into bimetallic Ag/Cu nanoparticles has demonstrated enhanced and synergistic properties. Thirumoorthy et al. reported that Ag/CuNPs exhibit potent antibacterial and antioxidant activities, making them strong candidates for combating biofilms and exploring novel therapeutic interventions [5].

Nanoparticles can be synthesized through various methods, broadly classified into top-down and bottom-up approaches. These approaches are further divided into different subclasses based on the method, reaction condition, and adopted protocols. Top-down synthesis, such as lithography, mechanical milling, sputtering, and laser ablation, involves breaking down bulk materials into NPs. Bottom-up synthesis, including laser pyrolysis, spinning, atomic or molecular condensation, and chemical reduction, involves building nanoparticles atom by atom [6]. The chemical reduction method uses agents such as citric acid or NaBH_4_ for the reduction of metal ions and polymers such as PEG for the stabilization of the particles [7]. These methods are costly and raise environmental and health risks, due to the chemicals that are used during synthesis.

To address these concerns, green synthesis methods using microorganisms and plant extracts have emerged as eco-friendly alternatives [1,8,9]. These approaches utilize the natural reducing and stabilizing agents in plants such as sugars, terpenoids, polyphenols, alkaloids, phenolic acids, and proteins to synthesize NPs while minimizing environmental impact [10,11].

Although plant extracts have been widely studied as reducing agents for NP synthesis, the use of *Cistus* extract remains underexplored. This extract is traditionally valued in herbal medicine for its therapeutic properties; *C. ladanifer*, for instance, has been used to treat conditions such as diarrhea, dysentery, catarrh, and menstrual pain [12]. Recent studies have highlighted its antioxidant, antibacterial, and antifungal properties [13]. For instance, Florkiewicz et al. used *Cistus incanus* L. extract to synthesize AgNPs, with cytotoxicity, immune combability, and antibacterial efficacy [14]. Additionally, Brindhadevi et al. functionalized AgNPs with *C. ladanifer* oil, known as labdanum oil, in order to improve the wound healing capacity of wound dressing fabrics [15]. Jing et al. also synthesized CuONPs using *C. incanus* L. leaf extract to assess their potential effects on alloxan-induced cardiac injury in rats, and Düz et al. synthesized AgNPs with *C. creticus* L. extract using the microwave-assisted method [16,17]. Despite these promising findings, metallic and bimetallic nanoparticles synthesized directly using *C. creticus* aqueous extract as a reducing agent remain largely unexplored, and a simple, fast, effective, and economical method has yet to be employed. To address this gap, this study combines the antimicrobial and antioxidant properties of Ag and Cu with the bioactive compounds of *C. creticus* extract, to develop functional nanomaterials for pharmaceutical and medical applications.

This work presents a comparative investigation of AgNPs, CuONPs, and bimetallic Ag/CuONPs, synthesized using *C. creticus* L. extract. The synthesized NPs were characterized using dynamic laser scattering (DLS), electrophoretic laser scattering (ELS), ultraviolet–visible spectrophotometry (UV-Vis), X-ray diffraction (XRD), total reflection X-ray fluorescence (TXRF), attenuated total reflectance (ATR), and transmission electron microscopy (TEM). Furthermore, the antioxidant activity of the extract and synthesized NPs was determined by evaluating the scavenging activity of the 2,2-diphenyl-1-picrylhydrazyl (DPPH) radical.

## 2. Results

### 2.1. Synthesis of NPs Monitored by Spectrophotometry

**Figure 1:**
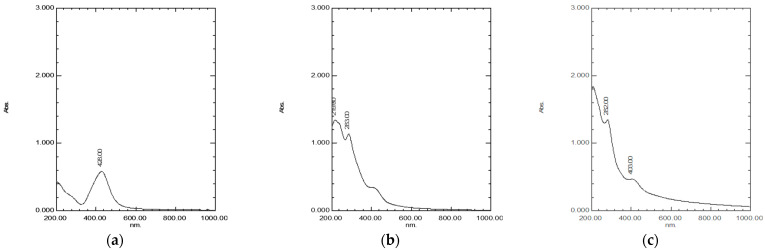
UV-Vis spectra and absorbance peaks of (**a**) AgNPs, (**b**) CuONPs, and (**c**) Ag/CuONPs synthesized using *C. creticus* L. extract.

The synthesis of AgNPs, CuONPs, and Ag/CuONPs using *C. creticus* L. extract was confirmed using UV-Vis spectroscopy. The UV-Vis spectrum of *C. creticus* L. extract showed characteristic absorption peaks at 266 and 362 nm (Figure S1). The synthesized AgNPs displayed an absorbance peak at 428 nm, while the CuONPs exhibited peaks at 219 and 283 nm. For the bimetallic Ag/CuONPs, absorbance peaks were observed at 282 and 403 nm. The results are in agreement with previous works [18,19,20,21,22]. The UV-Vis spectra of synthesized NPs are depicted in Figure 1.

### 2.2. Optimization of Experimental Parameters for Nanoparticle Synthesis

#### 2.2.1. Influence of Metal Precursor Concentration on Synthesis of NPs

The optimum conditions for the synthesis of each type of metal NP were first assessed by investigating the concentration of the metal precursors. Initially, solutions of 1 M and 2 M of AgNO_3_ and CuCl_2_·2H_2_O were prepared. Following the reaction and reduction process, the resulting particle sizes exceeded 600 nm (Table S1). These findings highlighted the necessity of reducing the concentration of the metal precursors to achieve smaller particle sizes.

#### 2.2.2. Role of Extract Concentration in Synthesis of NPs

Various concentrations of the *C. creticus* L. extract solution in water were prepared, ranging from 0.02 to 1% (*w*/*v*). The results indicated that the optimum extract concentration was 0.1% (*w*/*v*). At this concentration, NPs with the smallest particle size were consistently obtained across all cases (Table S2).

#### 2.2.3. Imact of pH on Synthesis of NPs 

The influence of pH was the next parameter to be assessed. For AgNPs, basic pH (12) conditions failed to yield the characteristic UV-Vis absorbance peak at 420–480 nm, and no color change occurred, indicating the absence of AgNP formation.

For CuONPs, acidic pH (4–5) conditions also resulted in no color change in the reaction mixture, demonstrating that the copper ions were not reduced. However, under basic conditions (pH = 12), a distinct color change was observed, and UV-Vis spectrophotometry confirmed the successful synthesis of CuONPs.

In the case of Ag/CuONPs, the reaction at basic pH did not result in a color change, suggesting that the reduction of AgNO_3_ and CuCl_2_ was unsuccessful. On the contrary, when the reaction was conducted at acidic pH, the synthesis of Ag/CuONPs was confirmed through a noticeable color change in the solution (Figure S2).

#### 2.2.4. Influence of Stirring Time on Synthesis of NPs

The next step was to test the effect of stirring time. The stirring time was initially monitored in the range of 1–24 h. It was observed that not all reaction times led to NP formation. Specifically, for AgNPs, the optimal reaction time was 24 h according to the results from UV-Vis spectroscopy. For CuONPs, the 24 h reaction led to the oxidation of copper. Hence, the optimal stirring times were determined to be 1 h for CuONPs and 2 h for Ag/CuONPs (Figure S3).

### 2.3. Physicochemical Characterization of NPs

#### 2.3.1. Size and ζ-Potential

The mean size, PDI, and ζ-potential of NPs immediately after their synthesis were monitored by DLS or ELS, respectively, and are summarized in Table 1.

Among the nanoparticles, AgNPs exhibited the smallest size (77.3 ± 1.26 nm), while CuONPs had the largest particle size (238.0 ± 0.60 nm, *p* < 0.05). The size of bimetallic Ag/CuONPs was measured to be 127.0 ± 0.60 nm. AgNPs and CuONPs displayed similar PDI values (*p* > 0.05), indicating uniformity in particle size distribution. Additionally, the absolute value of ζ-potential for all nanoparticles exceeded 20 mV, suggesting satisfactory long-term colloidal stability. Specifically, for Ag/CuONPs, the ζ-potential value was −77.9 ± 2.99 mV. The negative ζ-potential values can be attributed to functional groups such as -O^−^ from flavones and a carboxyl group, present on the nanoparticle surfaces due to the biomolecules originating from *C. creticus* L. extract [23].

#### 2.3.2. Colloidal Stability of NPs

**Figure 2:**
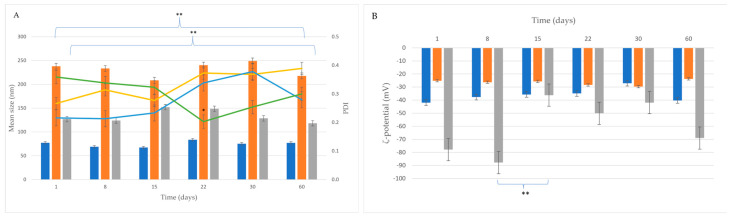
Stability study monitoring (**A**) size (bar), PDI (line), and (**B**) ζ-potential distribution of AgNPs (blue bar, yellow line), CuONPs (orange bar, blue line), and Ag/CuONPs (gray bar, green line) over time (* *p* < 0.05, ** *p* < 0.01).

The size and ζ-potential of AgNPs remained unchanged (*p* > 0.05) over 60 days of storage at room temperature, demonstrating good colloidal stability (Figure 2). On the other hand, CuONP size changed over the 60-day period (*p* < 0.01), possibly due to aggregation.

For Ag/CuONPs, the mean size did not change significantly during storage (*p* > 0.05). A tendency for decreased negative ζ-potential values was detected at storage times equal to or higher than 15 days (at 15 days, the change was statistically significant, *p* < 0.01); however, the system remained colloidally stable, showing no signs of aggregation over the studied storage period.

#### 2.3.3. Morphology of NPs

**Figure 3:**
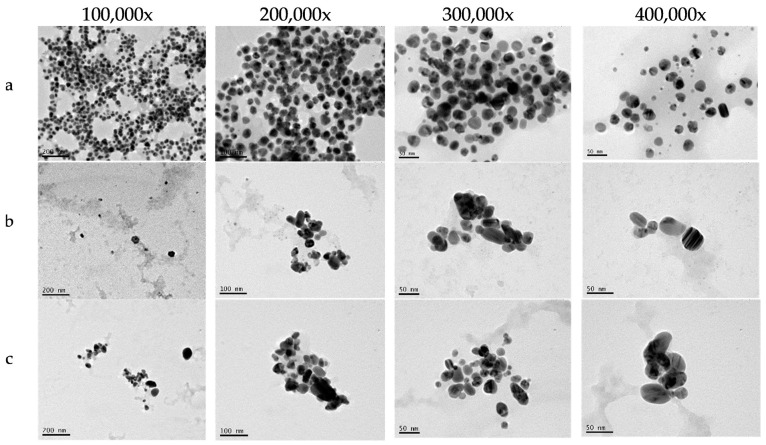
TEM images of AgNPs (**a**), CuONPs (**b**), and Ag/CuONPs (**c**) captured at magnifications of ×100,000, ×200,000, ×300,000, and ×400,000 magnitude. Scale bars represent 200 nm, 100 nm, and 50 nm, respectively.

The TEM images revealed the morphological characteristics of each type of metallic NP synthesized using *C. creticus* L. extract (Figure 3). AgNPs were spherical, non-agglomerated, and polydispersed, with particle sizes ranging from 12.4 to 60.5 nm. High-resolution TEM images of CuONPs and bimetallic Ag/CuONPs showed spherical and polymorphic particles of varying sizes. Additionally, lattice fringes were observed, indicative of a crystalline structure. The average diameter of CuONPs varied, with the smallest particles measuring 19.6 and the largest reaching 104.6 nm. In contrast, for Ag/CuONPs, the particle sizes ranged from 9.2 nm to 88.6 nm.

**Figure 4:**
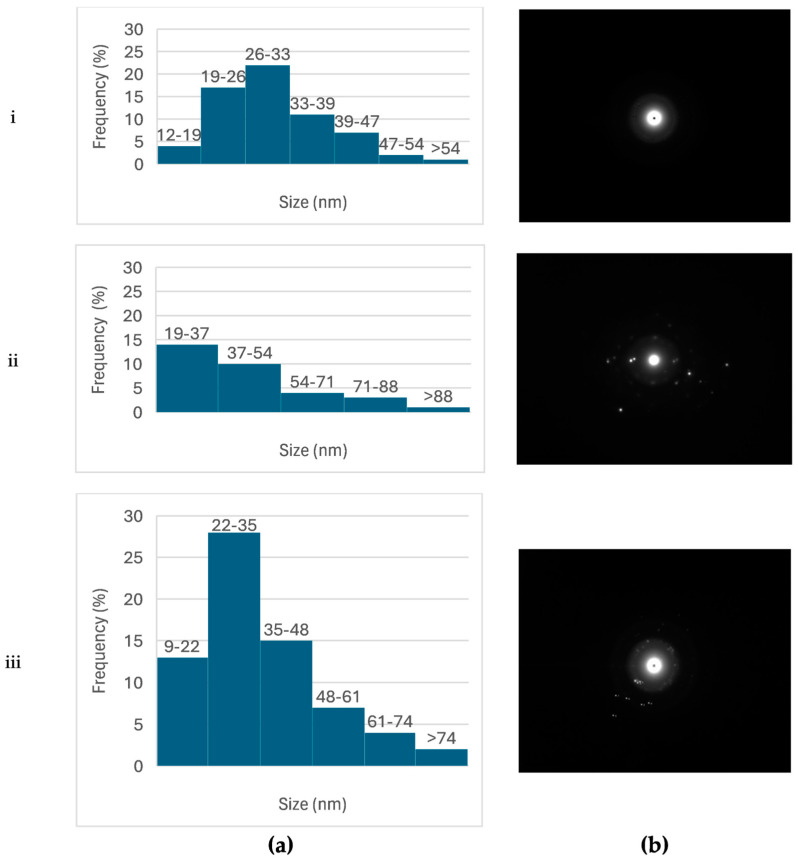
(**a**) Size distribution of AgNPs (**i**), CuONPs (**ii**), and Ag/CuONPs (**iii**) obtained from TEM image analysis. (**b**) Selected area electron diffraction (SAED) patterns of AgNPs (**i**), CuONPs (**ii**), and Ag/CuONPs (**iii**), also acquired through TEM, confirming their crystalline structure.

The particle sizes determined by TEM were generally smaller than those measured by DLS, particularly for CuONPs (Figure 4). This discrepancy can be attributed to the difference between the hydrodynamic diameter measured by DLS and the actual particle size observed in TEM, as DLS measurements include contributions from particle hydration.

The TEM images further showed that all metallic NPs were surrounded by a thick capping layer, visible as a translucent halo around the particles.

#### 2.3.4. Crystallinity of NPs

The d-spacing between the lattices of AgNPs was measured using the acquired selected area electron diffraction (SAED) patterns (Table S3). The diffraction ring patterns and d-spacing values were associated with the typical lattice planes of the polycrystalline structure and verified the synthesis of AgNPs. The values for lattice planes were (111), (200), (220), and (311), are indicative of a face-centered cubic (FCC) structure. XRD analysis further validated the crystalline characteristics of the obtained AgNPs, revealing peaks at 2*θ* values of 38.10°, 44.37°, 64.18°, and 77.5°, which correspond to the (111), (200), (220), and (311) planes, respectively (Figure S5). These results were in accordance with those from SAED patterns.

XRD analysis for CuONPs showed peaks at 2*θ* values of 29.36°, 36.50°, 42.19°, 61.35°, and 69.58°, corresponding to the (110), (111), (200), (220), and (310) planes, respectively (Figure S5). The results indicated a cubic cuprite phase (JCPDS Card No. 01-077-0199), and the results were further corroborated by SAED patterns. Scherrer’s equation was applied to estimate the mean crystallite size of CuONPs, yielding a value of 20.3 nm.

For bimetallic Ag/CuONPs, their successful formation was confirmed using XRD analysis. The appearance of peaks at 2*θ* values of 17.61°, 28.09°, 30.61°, and 43.82°, corresponding to the (101), (110), (200), and (220) planes, respectively, is indicative of the body-centered tetragonal structure of Cu_4_O_3_ and verifies the presence of CuO. Additional peaks at 44.35°, 52.10°, and 59.68° corresponding to the (200), (113), and (210) planes, respectively, suggest the hexagonal structure of Ag_2_O and verify the presence of Ag. These findings are in agreement with the SAED patterns (Table S3), where distinct diffraction rings corresponded to the crystallographic planes of both Ag and CuO phases.

#### 2.3.5. Chemical Composition of NPs

Elemental analysis (TXRF) confirmed the presence of silver (Ag) in AgNPs and bimetallic Ag/CuONPs, as well as copper (Cu) in CuONPs and Ag/CuONPs. The concentrations of Ag and Cu in the NP samples, obtained under optimum synthesis conditions, are summarized in Table 2.

The functional groups that participated in the reduction of Ag and Cu ions or in the stabilization of the synthesized AgNPs, CuONPs, and Ag/CuONPs were analyzed using attenuated total reflectance–Fourier-transform infrared spectroscopy (FTIR/ATR).

**Figure 5:**
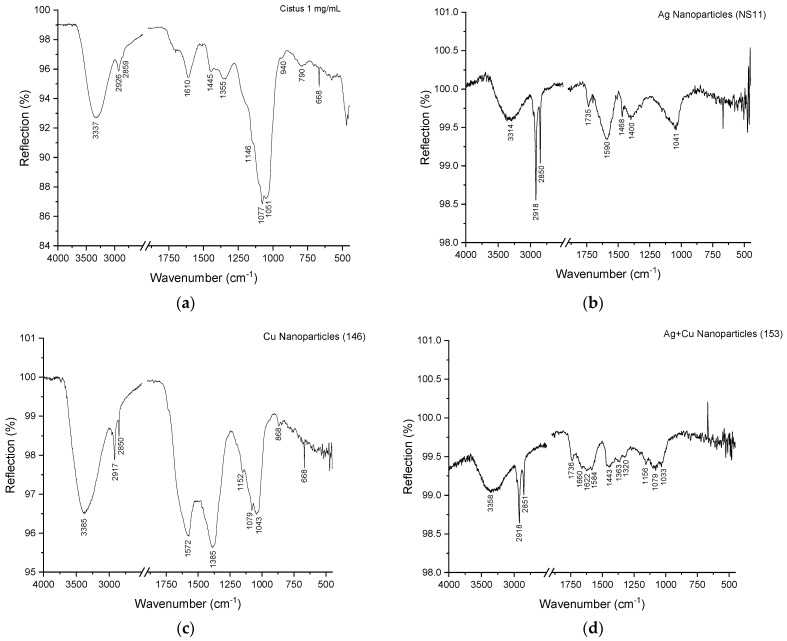
ATR spectra of (**a**) *C. creticus* L. extract, (**b**) AgNPs, (**c**) CuONPs, and (**d**) Ag/CuONPs.

The recorded spectra, spanning the 4000–500 cm^−1^ range, revealed several characteristic bands (Figure 5). The O-H stretching vibrations v(OH), observed in all spectra at approximately 3400 cm^−1^, were prominent and attributed to alcohols, phenols, carbohydrates, or water [7,24,25]. Shifts to lower wavenumbers in the spectra of the extract and NPs suggested the possible presence of an amine (N-H) bond [26]. The C-H stretching vibrations v(C-H), detected at around 2900 cm^−1^, indicated the presence of predominantly aliphatic C-H bonds [27]. A band at 1610 cm^−1^ was observed in the spectra of *C. creticus* L. extract and was attributed to the C=O group of carboxylic acids (COOH) or the N-H bond of primary amines [26].

The aromatic content of the samples was indicated by weak bands in the 1600–1450 cm^−1^ region, corresponding to the aromatic ring C=C stretching vibrations [25,27].

A band at 1445 cm^−1^, evident in *C. creticus* L. extract spectra, was assigned to CH_2_ scissoring vibration in the planar region [26].

Bands ca. 1100 cm^−1^ assigned to a C-H in-plane deformation mode and 1046 cm^−1^ assigned to C-O stretching vibrations from reducing sugars like glucose, fructose, and sucrose were prominent in the NPs, implying the involvement of these molecules in the reduction of Ag and Cu ions [28].

Finally, weak bands below 1000 cm^−1^ were attributed to various C-H out-of-plane deformations [23,24,27,29].

### 2.4. Antioxidant Activity

**Figure 6:**
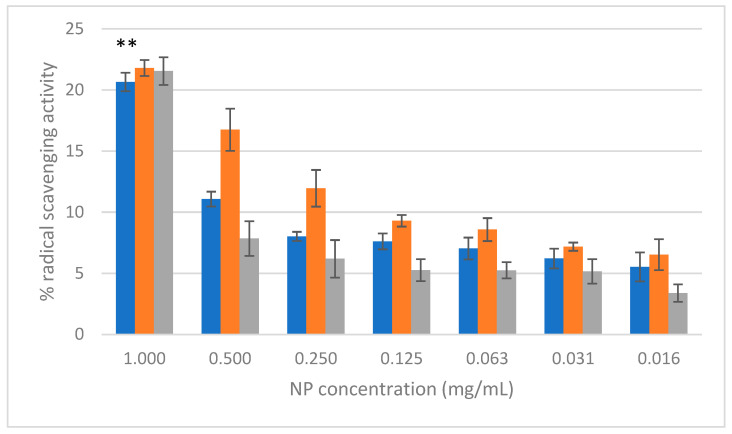
Antioxidant activity of AgNPs (blue), CuONPs (orange), and Ag/CuONPs (gray) as percentage of DPPH free radical inhibition (** *p* < 0.01).

The free radical scavenging activity of AgNPs, CuONPs, and Ag/CuONPs was evaluated using the DPPH assay, as illustrated in Figure 6. This activity was measured as the percentage of free radical scavenging across seven different concentrations (1.00 to 0.016 mg/mL).

CuONPs exhibited the highest apparent antioxidant activity across all concentrations, with a free radical scavenging activity of 21.8 ± 0.7% at 1 mg/mL. However, it is important to note that CuO is conventionally recognized as an oxidizing agent, and its measured antioxidant activity in this assay may be influenced by interactions with phytochemicals from the *Cistus creticus* extract, which could act as reducing agents or form complexes with Cu^2+^ ions. AgNPs displayed slightly lower scavenging activity than CuONPs, with a value of 20.7 ± 0.7% at the highest concentration. Ag/CuONPs showed comparable activity to CuONPs, achieving 21.5 ± 1.1% at 1 mg/mL. The *C. creticus* L. extract demonstrated the highest antioxidant activity overall, reaching 95.5 ± 0.8% at 2 mg/mL, although its activity dropped to 2.5 ± 0.6% at the lowest concentration (0.005 mg/mL). When comparing the calculated scavenging activity for the highest concentration (1 mg/mL) of synthesized NPs, the differences observed between AgNPs and CuONPs were statistically significant (*p* < 0.01), while those between AgNPs and CuONPs or between CuONPs and Ag/CuONPs were not statistically significant (*p* > 0.05).

Ascorbic acid, used as a positive control, achieved 98.7 ± 0.3% scavenging activity at 2 mg/mL and 7.1 ± 2.0% at 0.005 mg/mL.

## 3. Discussion

The synthesis of nanoparticles (NPs) using *Cistus creticus* L. extract represents an environmentally friendly and cost-effective approach to obtaining metallic and bimetallic nanoparticles with potential applications in pharmaceuticals and cosmetics. This study systematically optimized key reaction parameters, including the precursor concentration, extract concentration, pH, and reaction time, to evaluate their impact on nanoparticle synthesis and properties.

Plant extracts play a crucial role as reducing and stabilizing agents in NP synthesis. Specifically, for silver nanoparticles, when the extract is mixed with a silver ion solution (from silver nitrate), bioactive compounds such as proteins, polysaccharides, and phenolic compounds conduce to the reduction of Ag^+^ to metallic silver (Ag^0^) and stabilize the synthesized nanoparticles, protecting them from aggregation. The polarity of the composition environment significantly affects the characteristics of the NPs. Polar environments increase crystallinity, reactive oxygen species (ROS) production, and cytotoxicity, whereas non-polar environments result in lipid-coated nanoparticles with lower surface activity and reduced cytotoxicity [30].

The concentration of metal precursors played a critical role in nanoparticle formation and stability. Relatively high concentrations of AgNO_3_ and CuCl_2_·2H_2_O (1 M and 2 M) led to the formation of particles exceeding 600 nm in size, which are not suitable for pharmaceutical and cosmetic applications (Table S1). As reported in previous studies, reducing the precursor concentration to 10 mM resulted in smaller and more uniform particles [18,31,32,33]. Similarly, the extract concentration significantly influenced particle size, with 0.1% (*w*/*v*) being identified as the optimal concentration for all NPs. Higher extract concentrations led to larger particles, likely due to an excess of bioorganic molecules, which could cause uncontrolled aggregation [34,35].

The pH conditions were crucial in determining synthesis outcomes [36]. According to Siakavella et al. [18], the synthesis of AgNPs does not require pH adjustment. Consequently, AgNPs were synthesized here without pH adjustment at a specific pH value. It should be mentioned, however, that synthesis at basic pH (12) was also conducted, without the formation of NPs; therefore, basic pHs were avoided in AgNP syntheses. In the case of CuONPs, a highly basic pH (pH = 12) was necessary to facilitate the hydrolysis and subsequent formation of CuO. At high pH, Cu^2+^ ions undergo hydrolysis, leading to the precipitation of Cu(OH)_2_, which then transforms into CuO through dehydration. This process aligns with the thermodynamic equilibria of the Cu/H_2_O system, as described in the Pourbaix diagram [37]. These conditions were crucial for obtaining stable CuONPs, consistent with findings from previous research [38,39]. In contrast, the synthesis of bimetallic Ag/CuONPs was only successful under acidic conditions, with a notable color change confirming the formation of the nanoparticles. These findings highlight the need to tailor pH conditions based on the metal precursors and desired NP composition.

Reaction time also influenced the yield and quality of the NPs. For AgNPs, a 24 h reaction time under light and mild heating produced optimum results. CuONPs, on the other hand, required only 1 h of reaction time, as prolonged reaction times led to oxidation and reduced stability. Bimetallic Ag/CuONPs reached completion within 2 h, as indicated by the stabilization of UV-Vis absorbance peaks. These findings emphasize the need for time-optimized protocols to minimize energy consumption and prevent undesirable side reactions.

UV-Vis spectroscopy confirmed the successful synthesis of nanoparticles. The absorption peaks displayed by each type of metallic nanoparticle are consistent with earlier findings using different plant extracts [18,19,20,21,22]. For AgNPs, Düz et al. used *C. creticus* L. extract, reporting a characteristic resonance peak at 385.5 nm, and Ulusu et al. observed an absorbance peak at 517 nm using *Cistus salviifolius* L. extract, both differing from the peak at 428 nm found in this study [17,40]. CuONPs displayed absorption peaks at 219 nm and 283 nm, while Jing et al. refer to an absorption peak at 290 nm for CuONP synthesized with *C. incanus* leaf extract [16]. The shift in the absorption peak for silver ions to lower wavelengths can be attributed to variations in nanoparticle shape and agglomeration, as previously reported [41,42]. These spectral shifts confirm the reduction of metal ions and the formation of nanoparticles with unique optical properties.

Dynamic light scattering (DLS) and electrophoretic light scattering (ELS) revealed significant differences in size, the polydispersity index (PDI), and ζ-potential among the synthesized NPs. AgNPs exhibited the smallest size (77.3 ± 1.26 nm), while CuONPs were the largest nanoparticles (238.0 ± 0.60 nm). The bimetallic Ag/CuONPs had an intermediate size (127.0 ± 1.67 nm), reflecting the combination of the two metals. The ζ-potential values for all NPs exceeded ±20 mV, indicating satisfactory colloidal stability. Notably, Ag/CuONPs displayed the highest absolute ζ-potential (−77.9 ± 2.77 mV), suggesting a robust stabilizing effect of the capping agents derived from *C. creticus* L. extract. Ιn earlier findings, AgNPs from *C. creticus* L. extract had sizes ranging from 12.4 to 60.5 nm, while the average particle size of AgNPs from *C. salvifolius* L. extract was 12 nm [17,40]. For CuONPs, earlier studies showed particles with sizes between 15 and 25 nm, while ζ-potential and stability studies was not carried out [16]. The uniform particle size distribution, as indicated by the PDI, further supports the stability of the synthesized NPs.

Transmission electron microscopy (TEM) provided insights into the morphology and crystalline structure of the NPs. AgNPs were spherical and ranged in size from 12.4 to 60.5 nm, consistent with previous studies [16,43]. CuONPs and Ag/CuONPs exhibited polymorphic shapes, with some spherical particles interspersed, in agreement with previous studies [38,44]. TEM analysis confirmed that a significant proportion of CuONPs (19.6–104.6 nm) and Ag/CuONPs (9.2–88.6 nm) had at least one dimension within the nanoscale range (<100 nm). However, the hydrodynamic diameters measured by DLS were larger, with those for CuONPs reaching up to 238.9 nm and those for Ag/CuONPs up to 127 nm. These discrepancies can be attributed to the hydrodynamic diameter recorded by DLS, which accounts for the bioorganic capping layer and potential aggregation [45,46]. This stabilizing ligand layer is likely formed by organic molecules derived from the *C. creticus* L. extract that bind to the nanoparticle surface, increasing the apparent particle size in suspension. Additionally, the presence of this organic capping layer can influence the surface charge, potentially shifting the zeta potential and impacting the overall colloidal stability of the NPs [23].

Selected area electron diffraction (SAED) and X-ray diffraction (XRD) confirmed the crystalline nature of the NPs. AgNPs exhibited a face-centered cubic (FCC) structure, while CuONPs showed a cubic structure. For bimetallic Ag/CuONPs, the presence of CuO and Ag phases was confirmed, with distinct diffraction peaks corresponding to their respective lattice planes. These results align with previous studies demonstrating the crystalline characteristics of metallic and bimetallic nanoparticles [44].

The stability of the synthesized NPs was assessed over a 60-day period at room temperature. AgNPs remained highly stable, with no significant changes in size or ζ-potential, indicating minimal aggregation. CuONPs, however, showed evidence of aggregation, as reflected by an increase in particle size and a decrease in ζ-potential. Ag/CuONPs exhibited moderate stability, with minor aggregation observed after 15 days. The reduction in ζ-potential during this period suggests a partial loss of the capping layer, though the system remained relatively stable overall. These findings emphasize the importance of stabilizing agents and storage conditions in maintaining NP stability.

The antioxidant activity of the NPs was evaluated using the DPPH assay. CuONPs demonstrated the highest free radical scavenging activity (21.8 ± 0.7% at 1 mg/mL), followed closely by Ag/CuONPs (21.5 ± 1.1%) and AgNPs (20.7 ± 0.7%). The *C. creticus* L. extract exhibited superior antioxidant activity (95.5 ± 0.8% at 1 mg/mL), highlighting the potent bioactive compounds present in the extract. The apparent lower antioxidant activity of the NPs compared to the extract suggests that the bioactive compounds contribute more significantly to radical scavenging in their free form than when bound to NPs. These results suggest that the NPs have moderate antioxidant activity and that their primary value may lie in other applications, such as antimicrobial or drug delivery systems.

The use of *Cistus creticus* L. extract in the green synthesis of NPs provided multiple advantages. ATR spectroscopy confirmed the involvement of phenolic compounds, flavonoids, and reducing sugars in the reduction of metal ions and stabilization of the NPs. Although the exact composition of the commercially available extract used in this study is not fully disclosed by the manufacturer beyond a minimum total phenolic content of 20%, it is reasonable to hypothesize that phenolic compounds, such as flavonoids and tannins, act as key reductants for Ag^+^ ions. These compounds are well documented for their redox properties and ability to facilitate the biosynthesis of metal nanoparticles. Additionally, reducing sugars detected in ATR spectra may further contribute to the reduction process. The capping layer observed in TEM images, attributed to these bioorganic molecules, played a critical role in enhancing NP stability and preventing aggregation. This eco-friendly approach minimizes the environmental impact associated with conventional chemical synthesis methods and aligns with the principles of sustainable nanotechnology.

## 4. Materials and Methods

All the chemicals and solvents used were of analytical grade. Silver nitrate (Fisher Scientific, Leicester, UK), copper chloride dihydrate (Penta Chemicals Unlimited, Radiova, Prague, Czech Republic), 2,2-diphenyl-1-picrylhydrazyl (DPPH) (Sigma-Aldrich, Darmstadt, Germany), methanol (Fisher Scientific UK (Leicester, UK)), ascorbic acid (Sigma-Aldrich, Darmstadt, Germany), sodium hydroxide (Panreac Quimica SA, Barcelona, Spain), and phosphate-buffered saline (PBS) (Sigma-Aldrich, Darmstadt, Germany) were used. Distilled water was used in the entire study. Dried aqueous *Cistus* extract (*Cistus creticus* L. subsp. eriocephalus (viv.) synonym *Cistus incanus* subsp. Tauricus) (EXTR. CISTI E HERB. AQUOS. SICC. ECCE20, Martin Bauer Group, Vestenbergsgreuth, Germany) was donated by N.Krallis SA, Athens, Greece. According to the material specifications provided by the producers, this ingredient contains minimum 20% total polyphenols, and the composition used in extract preparation was 80% native extract and 20% excipients (18% maltodextrin Ph.Eur. and 2% silica, colloidal anhydrous Ph.Eur.).

### 4.1. Synthesis of NPs

The green synthesis of NPs was achieved using the *C. creticus* extract as the reducing agent. To optimize the reaction conditions, the impact of key parameters such as stirring time, pH, and extract and metal precursor concentrations were evaluated. These factors were analyzed for their influence on the yield and physicochemical characteristics of the synthesized NPs.

#### 4.1.1. Synthesis of AgNPs

The synthesis of AgNPs was achieved according to Siakavella et al. [18] by mixing a AgNO_3_ solution (10 mM) and a *C. creticus* extract solution (0.1% *w*/*v*) in a 3:2 volume ratio. The total volume of the reaction mixtures was 10 mL. The reaction mixtures were left under stirring for 24 h on a thermal magnetic stirrer at 50–60 °C [18]. After the formation of AgNPs, the solution was centrifuged at 12,000 rpm for 20 min. The resulting sediment was collected, dispersed in distilled water, and subjected to two additional washing cycles. The final dispersions were stored in falcon centrifuge tubes at room temperature for further analysis (Figure S4).

#### 4.1.2. Synthesis of CuONPs Using *Cistus creticus* L. Extract

A solution of CuCl_2_·2H_2_O (10 mM) and the *C. creticus L.* extract solution (0.1% *w*/*v*) were combined in a 3:2 volume ratio (total volume 10 mL) to synthesize CuONPs. The pH of the reaction mixture was adjusted to 12 using a 10 M NaOH solution (10 μL) to achieve strongly basic conditions. The reaction mixture was stirred at 50–60 °C for 1 h using a thermal magnetic stirrer. The synthesis of CuONPs was confirmed by a visible color change in the reaction mixture, transitioning from dark green to dark brown. Following CuONP synthesis, the solution was centrifuged at 12,000 rpm for 20 min. The sediment was collected and dispersed in distilled water. This washing process was repeated 3 times, and the final dispersions were stored in falcon centrifuge tubes at room temperature for further analysis (Figure S4).

#### 4.1.3. Synthesis of Ag/CuONPs Using *Cistus creticus* L. Extract

Ag/CuONPs were synthesized by combining a AgNO_3_ solution (10 mM), a CuCl_2_·2H_2_O solution (10 mM), and a *C. creticus* L. extract solution (0.1% *w*/*v*) in a 3:3:4 volume ratio (total volume 10 mL). The reaction mixture was stirred at 50–60 °C for 2 h on a thermal magnetic stirrer. The successful synthesis of Ag/CuONPs was confirmed by a visible color change in the reaction mixture, transitioning from light brown to dark brown. After synthesis, the solution was centrifuged at 12,000 rpm for 20 min. The sediment was collected and dispersed in distilled water. The washing process was repeated 3 times, and the final mixed dispersions were stored in falcon centrifuge tubes at room temperature for further analysis (Figure S4).

### 4.2. Physicochemical Characterization

The average hydrodynamic diameter (mean size), polydispersity index (PDI), and ζ-potential of NPs were monitored by dynamic and electrophoretic laser spectroscopy (DLS, ELS), respectively (Malvern Instruments Ltd., Malvern, UK). For the physicochemical characterization of NPs, the dispersions obtained after centrifugation and leaching were further diluted in distilled water in a 1:6 volume ratio.

### 4.3. Monitoring of NP Synthesis by Spectrophotometry

The confirmation of nanoparticle synthesis was performed using UV-Vis spectroscopy (UV-1800 UV-Vis Spectrophotometer, SHIMADZU, Kyoto, Japan). The presence of an absorption peak at 420–480 nm, attributed to the surface plasmon resonance (SPR) of metallic silver, confirmed the synthesis of AgNPs. Similarly, the peak observed at 220–350 nm verified the presence of CuONPs [19,20,21]. The existence of absorption peaks in both wavelength ranges suggested the successful formation of bimetallic Ag/CuONPs [22]. The absorption spectrum of *C. creticus* extract was analyzed to identify any peaks that might interfere with the identification of NPs.

### 4.4. Study of Experimental Parameters for Optimization of Nanoparticle Synthesis

#### 4.4.1. Effect of Metal Precursor Concentration

The effect of metal precursor concentration on NP synthesis was investigated using three different concentrations (1 M, 2 M, 10 mM). Solutions of AgNO_3_ and CuCl_2_·2H_2_O were prepared by dissolving the appropriate amounts of each salt in distilled water. The metal precursor solutions were mixed with *C. creticus* L. extract in volume ratios of 3:2 for AgNPs and CuONPs, and 3:3:4 for Ag/CuONPs. DLS was used to measure the particle size (hydrodynamic diameter) of the resulting NPs, and the concentration that produced the smallest and most stable particles was considered optimal.

#### 4.4.2. Effect of Extract Concentration

The aqueous extract of *Cistus creticus* L. was used as a reducing and capping agent. The extract was dissolved in distilled water to prepare solutions in various concentrations (0.02, 0.06, 0.1, 0.2, 0.5, and 1% (*w*/*v*)). To examine the influence of extract concentration, reactions were carried out using 10 mM of metal precursor and different concentrations of *C. creticus* L. each time in a specific volume ratio (3:2 for AgNPs and CuONPs and 3:3:4 for bimetallic NPs). The particle size of the synthesized NPs was measured by DLS to determine the optimal extract concentration for producing stable nanoparticles with minimal aggregation.

#### 4.4.3. Effect of pH on Synthesis of NPs

The pH of reaction mixtures was initially measured using a pH meter (HI 207, Hanna Instruments, Woonsocket, Rhode Island, US) and found to be in the acidic range (pH = 4–5). Firstly, reactions were carried out without pH adjustment using 10 mM AgNO_3_ and/or CuCl_2_·2H_2_O and 0.1% (*w*/*v*) *C. creticus* L. extract in the specified volume ratio. Subsequently, the adjustment of pH to 12 was carried out using a 10 M NaOH solution, and the presence of NPs was confirmed through UV-Vis spectroscopy by observing characteristic absorption peaks.

#### 4.4.4. Effect of Stirring Time on Synthesis of NPs

The reaction mixtures were left under light at the ideal pH for 24 h, with heating at 50–60 °C. At predetermined time points (1, 2, 5, and 24 h), a sample of reaction mixture was tested for the presence of NPs spectrophotometrically, according to Siakavella et al. [18].

For CuONPs, a green-blue solution was observed after 2 h, indicating copper oxidation. To optimize synthesis conditions, the reaction was repeated under the optimal conditions for 1 h, with absorbance measurements taken at 15 min, 30 min, 45 min, and 60 min.

In the case of Ag/CuONPs, no change in absorption intensity was observed after 2 h of stirring. Consequently, a kinetic study was conducted for 2 h, with absorbance measurements taken at 30, 60, 90, and 120 min. The reaction completion time was defined as the point at which the absorption peak ceased to increase.

### 4.5. Stability Study of NPs

The colloidal stability of the NPs was evaluated by storing the dispersions at room temperature for a period of 60 days. The particle size, PDI, and ζ-potential were measured at predetermined time intervals (1, 8, 15, 22, 30, and 60 days), following the described methodology.

### 4.6. Morphological Characterization of NPs

The morphology of NPs was examined using transmission electron microscopy (TEM; JEOL JEM-2100, Tokyo, Japan). The particle size of the NPs was determined analyzing the TEM images using ImageJ version 1.54m 5 December 2024 [47], and size distribution graphs were constructed. Additionally, selected area electron diffraction (SAED) patterns were obtained, revealing the distances between diffraction centers and confirming the crystalline structure of NPs.

### 4.7. Structural, Elemental, and Functional Group Analysis

#### 4.7.1. X-Ray Diffraction (XRD)

The crystalline structure of the nanoparticles was characterized using X-ray diffraction (XRD) analysis. The measurements were performed with a Bruker D8 Advance Diffractometer (Bruker, Billerica, MA, USA) equipped with Ni-filtered CuKα radiation, operating at an accelerating voltage of 40 kV and a current of 40 mA. Prior to analysis, the nanoparticles were lyophilized to ensure the removal of residual moisture. XRD patterns were recorded over a 2θ range of 2–70° at a scanning step size of 0.015° and a scan speed of 0.1 s per step. Random powder mounts were prepared by gently pressing the lyophilized nanoparticle powder into the cavity holder. Phase identification was carried out using DIFFRAC plus EVA12^®^ software version 12 rev.0 (Bruker-AXS, Fitchburg, WI, USA) in conjunction with the ICDD Powder Diffraction File (PDF-2, 2006). The detection limit of the XRD analysis was approximately 2–3%.

#### 4.7.2. Total Reflection X-Ray Fluorescence (TXRF)

Elemental composition analysis was conducted through total reflection X-ray fluorescence spectroscopy (TXRF) using a benchtop spectrometer (S2 PICOFOX™, Bruker Nano GmbH, Berlin, Germany) equipped with a molybdenum (Mo) target X-ray tube operating at 30 W (50 kV, 0.6 mA). Gallium (Ga) was used as the internal standard, the total time of the analysis was set at 1000 s, and spectral data were processed using S2 PICOFOX™ software version 7.5.3.0 (Bruker Nano GmbH, Berlin, Germany).

For each measurement, the sample was mixed with Ga to a final concentration of 10 mg/L and 10 μL of the solution was placed on siliconized quartz sample holders. Subsequently, each carrier was heated at 50 °C for about 10 min on a heat plate until the solvent was evaporated completely and a thin film was formed. The process was repeated in triplicate for each sample tested.

#### 4.7.3. Attenuated Total Reflectance–Fourier-Transform Infrared Spectroscopy (ATR-FTIR)

To identify functional groups on the surface of the nanoparticles, FTIR/ATR spectra were recorded in the range of 4000–500 cm^−1^ using a PerkinElmer Spectrum 100 FTIR spectrometer (Waltham, MA, USA). An attenuated total reflectance (ATR) accessory with a single-bounce ZnSe diamond crystal was employed for sample analysis. Each spectrum was the sum of 20 accumulated scans with a spectral resolution of 4 cm^−1^.

For the ATR analysis of the samples, 5 μL of each sample was placed on the crystal to dry for approximately 30 min. After the evaporation of the solvents of the samples, their ATR spectra were acquired.

### 4.8. Evaluation of NPs for Antioxidant Activity

Free radical scavenging activity was assessed using the 2,2-diphenyl-1-picrylhydrazyl (DPPH) assay, following the methods described by Brand-Williams [48] and Kim et al. [49]. A 0.1 mM DPPH solution in methanol was prepared, and 195 μL of this solution was added to each well of a 96-well plate. Subsequently, 5 µL of varying concentrations (1 mg/mL, 0.5 mg/mL, 0.25 mg/mL, 0,13 mg/mL, 0.06 mg/mL, and 0.03 mg/mL) of *C. creticus* extract, AgNPs, CuONPs, Ag/CuONPs, or a standard solution was added to the DPPH solution. The final reaction mixtures were incubated at 25 °C for 30 min, protected from light. Following incubation, the absorbance of the reaction mixtures was measured at 540 nm using a Sunrise^TM^ microplate reader (TECAN Trading AG, Männedorf, Switzerland). Each sample was analyzed in triplicate.

The percentage DPPH radical scavenging activity was calculated using Equation (1):

DPPH radical scavenging% = 100 − (Abs(control) − Abs(sample))/Abs(control) × 100(1)

where Abs(sample) represents the absorbance of the reaction mixture containing the sample, and Abs(control) represents the absorbance of the DPPH solution without any sample.

Ascorbic acid was used as a positive control at concentrations ranging from 2 mg/mL to 0.005 mg/mL to validate the efficacy of the assay.

### 4.9. Statistical Analysis

The results are expressed as mean values ± standard deviation (SD) for triplicate measurements. Statistical significance was determined using a Student’s *t*-test performed with Microsoft Office Excel 2024 (Redmond, WA, USA).

## 5. Conclusions

This study demonstrates the successful green synthesis of silver nanoparticles (AgNPs), copper nanoparticles (CuONPs), and silver–copper nanoparticles (Ag/CuONPs) using *Cistus creticus* L. extract as a reducing and stabilizing agent. The optimization of synthesis parameters, including precursor concentration, extract concentration, pH, and reaction time, yielded nanoparticles with distinct physicochemical properties. AgNPs were the smallest and most stable, CuONPs exhibited the highest antioxidant activity, and Ag/CuONPs exhibited an intermediate size, stability, and antioxidant potential. The involvement of bioorganic compounds, confirmed by ATR spectroscopy, played a critical role in nanoparticle formation and stabilization, with stability maintained over 60 days. These findings indicate the potential of *Cistus creticus* L. extract in green metallic nanoparticle synthesis and point toward the eco-friendly and sustainable nature of green synthesis, offering a viable alternative to conventional methods. While AgNPs are ideal for applications requiring high stability, CuONPs show potential for antioxidant-related applications, and Ag/CuONPs present opportunities for multifunctional use in pharmaceuticals and cosmetics. These results also demonstrate the potential of *Cistus creticus* L. extract in nanoparticle synthesis, leading to novel materials with enhanced antimicrobial properties and a low potential of systemic toxicity.

## Figures and Tables

**Table 1:** Physicochemical characteristics of NPs (mean values ± SD).

Nanoparticles	Mean Size (nm)	PDI	ζ-Potential (mV)
AgNPs	77.3 ± 1.26	0.266 ± 0.012	−41.9 ± 2.65
CuONPs	238.0 ± 0.60	0.216 ± 0.009	−25.2 ± 1.63
Ag/CuONPs	127.0 ± 1.67	0.359 ± 0.012	−77.9 ± 2.77

**Table 2:** Concentration (mg/L) of Ag and Cu in NPs (mean values ± SD).

Sample	Ag (% *w*/*v*)	Cu (% *w*/*v*)
AgNPs	138.6 × 10^−4^ ± 16.5 × 10^−4^	0
CuONPs	0	111.5 × 10^−4^ ± 2.4 × 10^−4^
Ag/CuONPs	29.8 × 10^−4^ ± 0.9 × 10^−4^	19.2 × 10^−4^ ± 0.1 × 10^−4^

## Data Availability

Data are available upon request.

## Supplementary Materials

The following supporting information can be downloaded at https://www.mdpi.com/article/10.3390/ijms26062518/s1.

## Abbreviations

The following abbreviations are used in this manuscript:

Ag	silver
CuO	copper oxide
NPs	nanoparticles
ΤXRF	total X-ray fluorescence
DLS	dynamic light scattering
ELS	electrophoretic light scattering
TEM	transmission electron microscopy
SAED	selected area electron diffraction
XRD	X-ray diffraction
