# Supplementary material for: Green Synthesis and Comparative Analysis of Silver, Copper Oxide, and Bimetallic Ag/CuO Nanoparticles Using Cistus creticus L. Extract: Physicochemical Properties, Stability, and Antioxidant Potential
Source: Int J Mol Sci. 2025 Mar 11;26(6):2518. doi: 10.3390/ijms26062518 (PMC11941820; doi:10.3390/ijms26062518)
Supplement: Supplementary file 1 [file ijms-26-02518-s001.zip › ijms-3494671-supplementary.pdf]

Supplementary Materials

Green Synthesis and Comparative Analysis of Silver, Copper Oxide, and Bimetallic Ag/CuO Nanoparticles Using *Cistus creticus* L. Extract: Physicochemical Properties, Stability, and Antioxidant Potential

Chrysi Chaikali [1], Nicole Dora Stola [1], Paraskevi Lampropoulou [2], Dimitrios Papoulis [2], Fotini N. Lamari [3], Malvina Orkoula [4], Michail Lykouras [5] and Konstantinos Avgoustakis [1] and Sophia Hatziantoniou [1,*]

[1] Laboratory of Pharmaceutical Technology, Department of Pharmacy, School of Health Sciences, University of Patras, 26504 Patras, Greece; chem3813@ac.upatras.gr (C.C.); up1022689@upnet.gr (N.D.S.); avgoust@upatras.gr (K.A.)

[2] Department of Geology, University of Patras, 26504 Patras, Greece; p.lampropoulou@upatras.gr (P.L.); papoulis@upatras.gr (D.P.)

[3] Laboratory Pharmacognosy & Chemistry of Natural Products, Department of Pharmacy, School of Health Sciences, University of Patras, 26504 Patras, Greece; flam@upatras.gr

[4] Laboratory of Instrumental Pharmaceutical Analysis, Department of Pharmacy, School of Health Sciences, University of Patras, 26504 Patras, Greece; malbie@upatras.gr

[5] Institute of Chemical Engineering Sciences, Foundation of Research and Technology-Hellas (ICE-HT/FORTH), 26504 Patras, Greece; lykouras@iceht.forth.gr

* Correspondence: sohatzi@upatras.gr

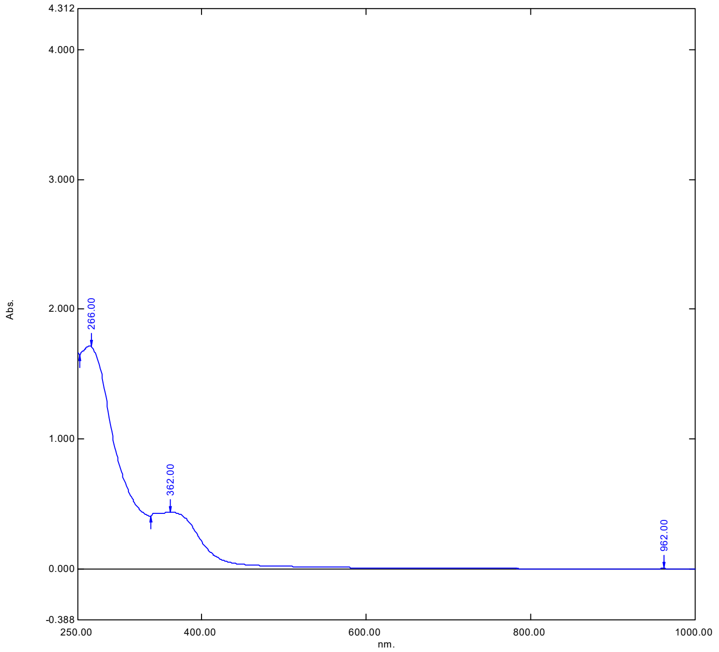

Figure S1. UV/Vis spectrum and absorbance peak of *Cistus creticus* L. extract.

Table S1. Effect of metal precursor concentration (1 and 2 M) on the physicochemical characteristics of NPs.

Nanoparticle	Concentration of Metal Precursor (M)	Mean Size (nm)	PDI	ζ-Potential (mV)
AgNPs	1	616.30 ± 12.46	0.165 ± 0.047	-28.97 ± 0.529
	2	852.71 ± 41.35	0.312 ± 0.037	-31.63 ± 2.32
CuONPs	1	949.12 ± 83.23	0.395 ± 0.091	-23.12 ± 0.636
	2	1005 ± 216.20	0.352 ± 0.196	-14.88 ± 0.651
Ag/CuONPs	1	865.30 ± 233.41	0.553 ± 0.080	-25.40 ± 2.98
	2	2037 ± 188.8	0.563 ± 0.064	-17.00 ± 0.40

Table S2. Effect of *Cistus creticus* L. extract concentration on the physicochemical characteristics of NPs.

Nanoparticle	*Cistus creticus* L. Extract Concentration (w/v)%	Mean Size (nm)	PDI	ζ-Potential (mV)
AgNPs	1	480.30 ± 1.84	0.324 ± 0.044	-28.60 ± 0.76
	0.5	141.12 ± 1.72	0.336 ± 0.020	-34.10 ± 1.95
	0.2	77.30 ± 0.91	0.279 ± 0.039	-35.00 ± 1.20
	0.1	65.78 ± 1.26	0.266 ± 0.012	-41.87 ± 2.65
	0.06	103.40 ± 2.63	0.526 ± 0.112	-44.50 ± 2.45
	0.02	112.60 ± 8.13	0.407 ± 0.062	-28.00 ± 3.72
CuONPs	1	751.02 ± 24.15	0.450 ± 0.055	-29.43 ± 1.35
	0.5	302.43 ± 5.77	0.334 ± 0.036	-26.40 ± 0.35
	0.2	253.17 ± 3.11	0.270 ± 0.009	-27.86 ± 0.62
	0.1	238.03 ± 0.60	0.216 ± 0.009	-25.23 ± 1.63
	0.06	305.38 ± 2.58	0.249 ± 0.024	-21.46 ± 2.46
	0.02	423.59 ± 6.46	0.352 ± 0.031	-19.34 ± 5.74
Ag/CuONPs	1	223,77 ± 1.52	0,264 ± 0.017	-32,4 ± 5.82
	0.5	227,65 ± 8.49	0,214 ± 0.004	-45,8 ± 1.42
	0.2	167,30 ± 4.18	0,352 ± 0.035	-23,7 ± 0.59
	0.1	127.01 ± 1.67	0.359 ± 0.012	-77.90 ± 2.77
	0.06	148.63 ± 3.40	0.203 ± 0.013	-50.10 ± 1.41
	0.02	338.3 ± 32.39	0.534 ± 0.076	-42.5 ± 0.36

Table S3. SAED patterns of NPs.

d-spacing (nm)	
AgNPs	Miller index
0.2385<br>0.2043<br>0.1415<br>0.1244	Ag(111)<br>Ag(200)<br>Ag(220)<br>Ag(311)
CuONPs	
0.2506<br>0.2357<br>0.2072<br>0.1664	CuO(110)<br>CuO(111)<br>CuO(200)<br>CuO(113)
Ag/CuONPs	
0.2587<br>0.2376<br>0.2128<br>0.2070<br>0.1806<br>0.1664<br>0.1267	CuO(110)<br>Ag(111)<br>CuO(200)<br>Ag(200)<br>CuO(202)<br>Ag_2O(220)<br>Ag(311)

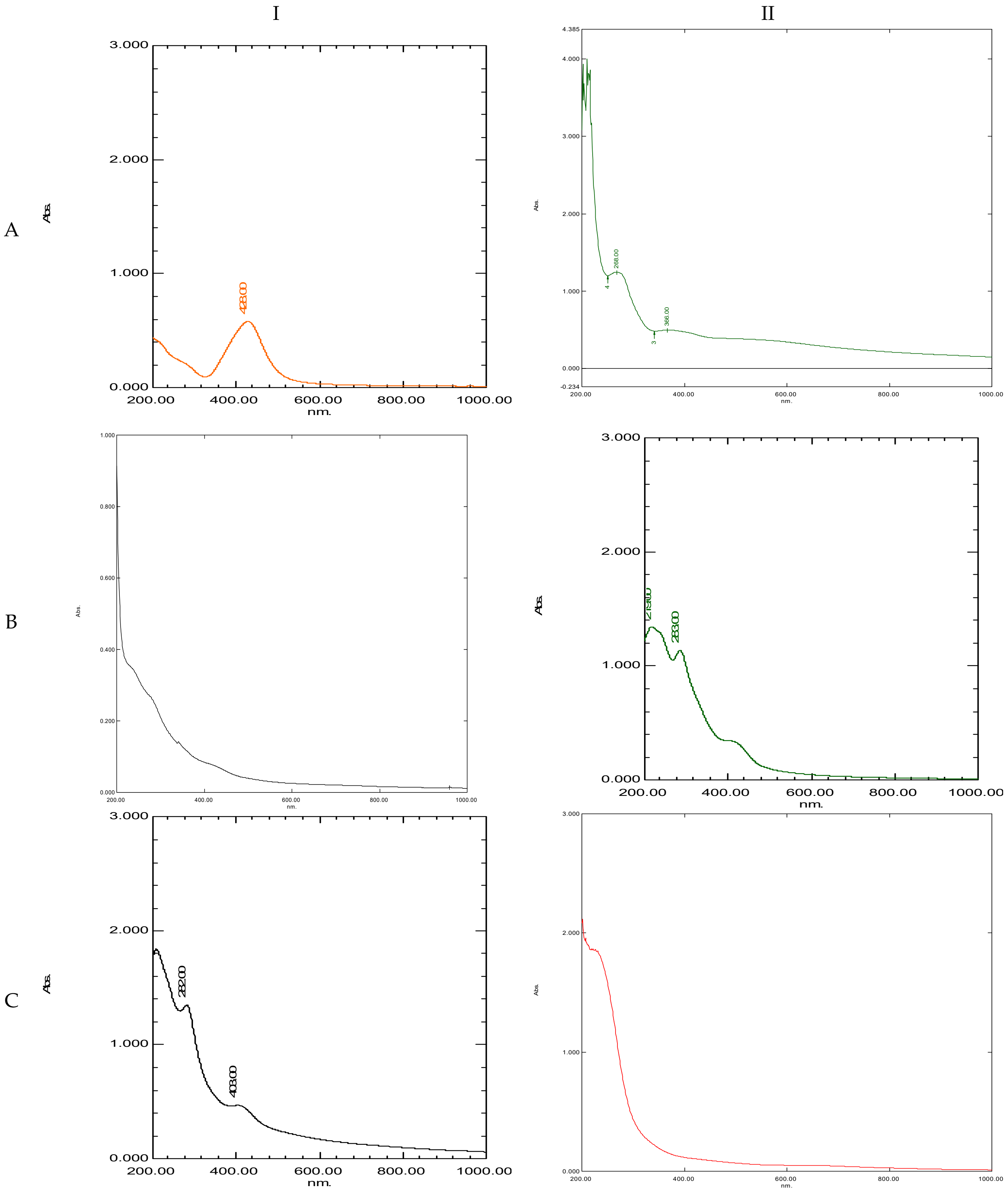

Figure S2. Effect of pH on the synthesis of AgNPs (**A**), CuONPs (**B**) and Ag/CuONPs (**C**), without (I) and with (II) pH adjustment).

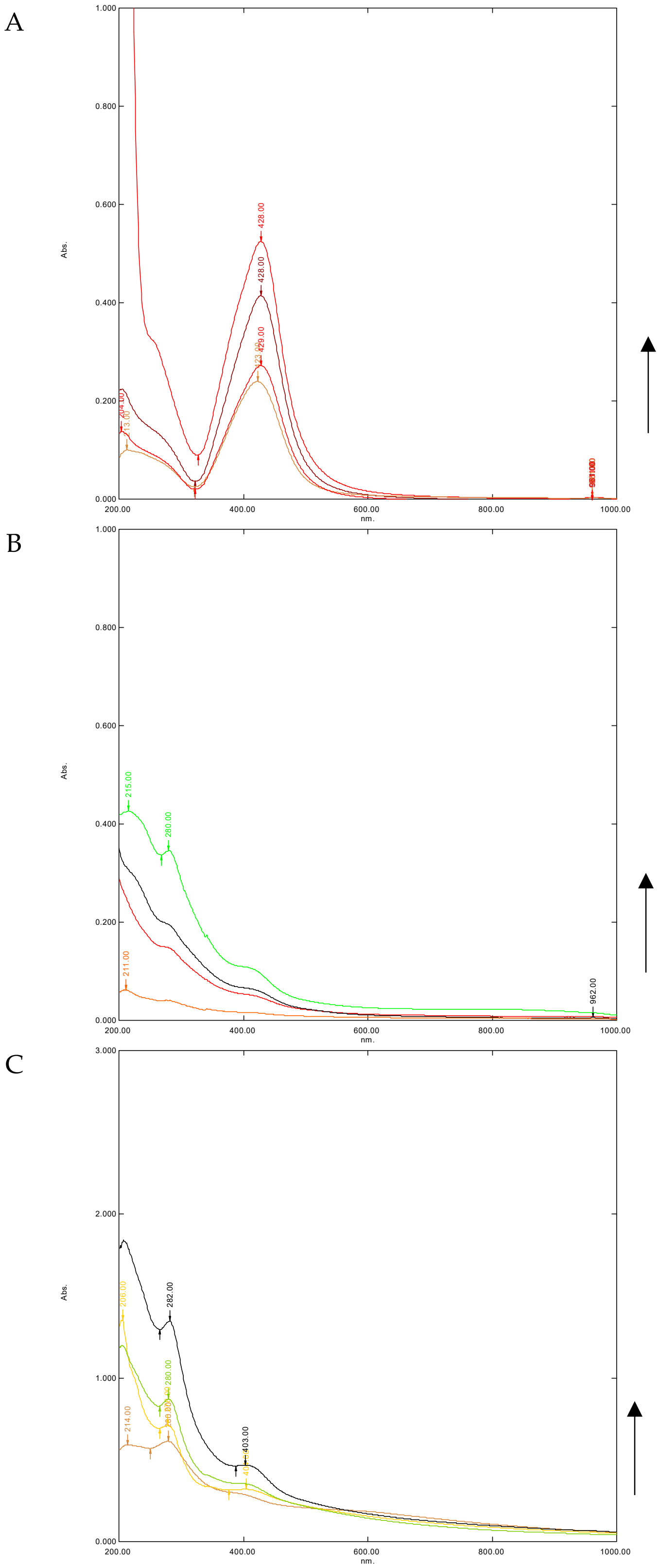

Figure S3. Study of the synthesis of AgNPs (**A**), CuONPs (**B**) and Ag/CuONPs (**C**) monitoring the intensity of the absorbance of the reaction mixture.

A

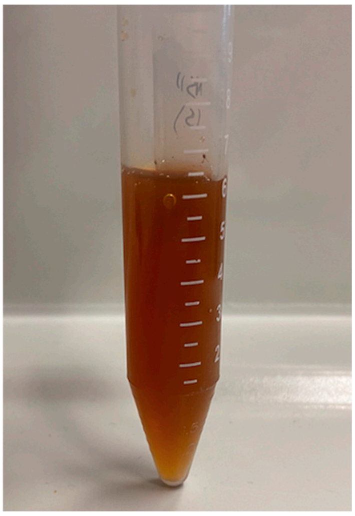

B

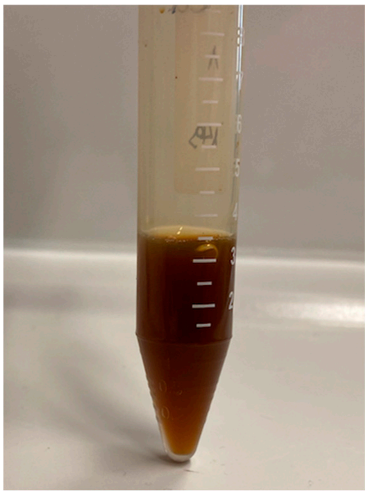

C

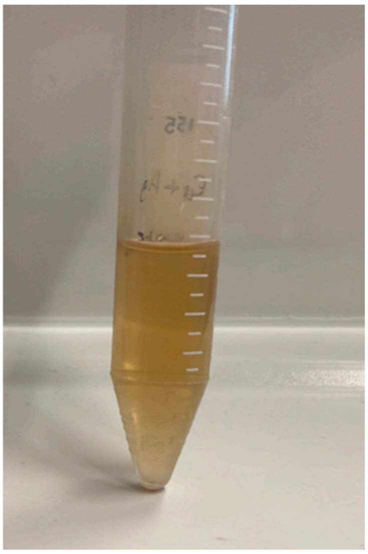

Figure S4. Photographs of the synthesized AgNPs (**A**), CuONPs (**B**) and Ag/CuONPs (**C**).

A

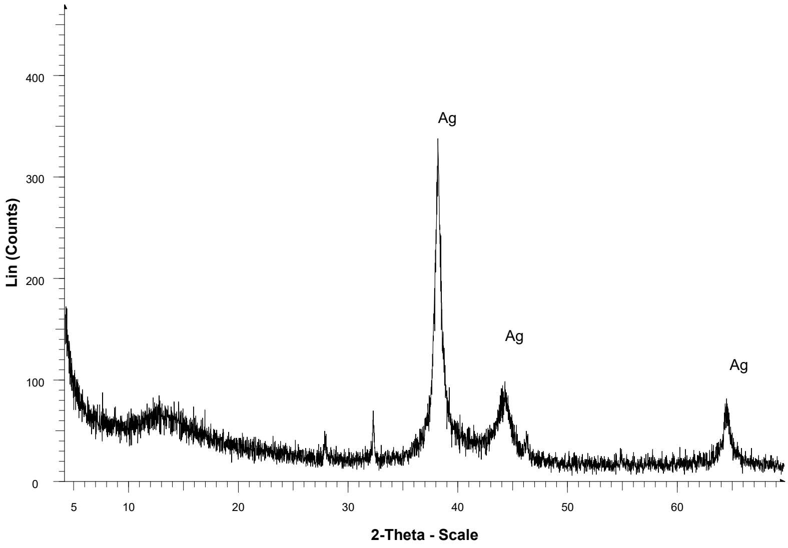

B

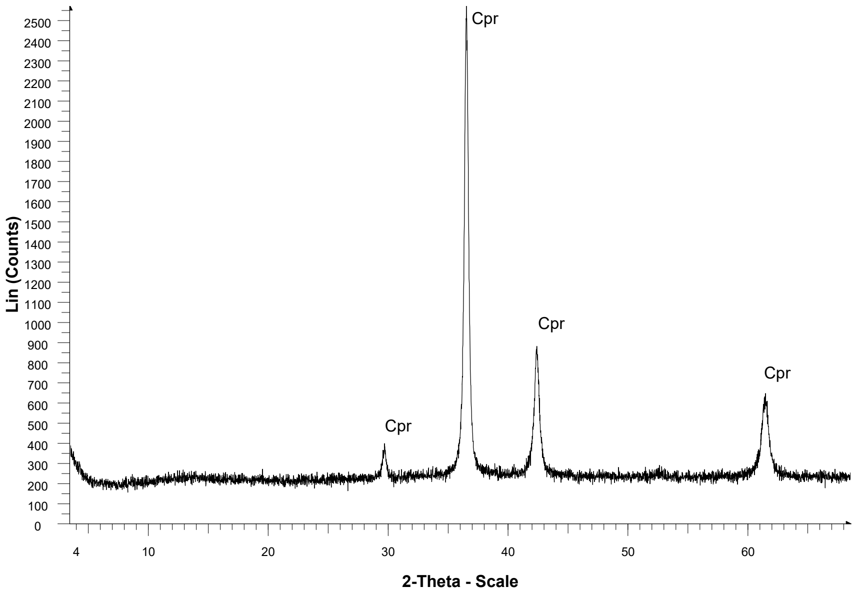

Figure S5. XRD spectra of AgNPs (**A**), CuONPs (**B**) and Ag/CuONPs.
